# Reply to the Editor Regarding: Magnusson MR, Deva AK Letter to the Editor 2018 May 2 in Relation to: Fleming D, Stone J, Tansley P. Spontaneous Regression and Resolution of Breast Implant-Associated Anaplastic Large Cell Lymphoma: Implications for Research, Diagnosis and Clinical Management. Aesth Plast Surg, 2018

**DOI:** 10.1007/s00266-018-1169-z

**Published:** 2018-06-11

**Authors:** Daniel Fleming, Jason Stone, Patrick Tansley

**Affiliations:** 1 Cosmetic Surgery Institute of Australia, P.O. Box 213, Fortitude Valley, Brisbane, QLD 4006 Australia; 2 QML Pathology, 1 Riverview Place, Metroplex on Gateway, Murarrie, QLD 4172 Australia; 3 NorthEast Plastic Surgery, Wickham House, Level 1 155 Wickham Terrace, Spring Hill, Brisbane, QLD 4000 Australia

*Level of Evidence V* This journal requires that authors assign a level of evidence to each article. For a full description of these Evidence-Based Medicine ratings, please refer to the Table of Contents or the online Instructions to Authors www.springer.com/00266.

Dear Sir,

In their letter [1] about our paper [2], we note that Drs. Magnusson and Deva have not recognized its central hypothesis. They may have failed to appreciate the interpretation and significance or otherwise of the two cases described is entirely predicated on the epidemiological analysis that precedes it. We first identified the epidemiological evidence presented, together with its implications, some 3 years ago. Australia’s Chief Medical Officer and the Therapeutic Goods Administration were alerted to this evidence in June 2015 [3] by the first author who also provided details of it to Dr. Deva in June 2016 [4]. Nevertheless, Drs. Magnusson and Deva, like Drs. Miranda and Clemens before them [5], have missed the epidemiology detailed in the paper.

We refer Drs. Magnusson and Deva to our detailed response to Drs. Miranda and Clemens’ letter [6] and would emphasize the importance of assessing the totality of the evidence before dismissing new findings.

Drs. Magnusson and Deva then make various claims which are not supported by facts or what the paper actually states.

## **Claim 1**

“Dr Miranda reports in his response that there was demonstrable tumor still present—albeit after a more detailed pathological examination at multiple time points in the patient’s history.”

## **Fact**

The “detailed pathological examination at multiple time points in the patient’s history” had already been performed by us prior to submitting the slides to Dr. Miranda; his findings entirely concurred with those we had already made and were accurately reported in the paper. This included comment that confirmed “there was demonstrable tumor still present,” albeit a > 95% reduction, which is precisely why this case was considered as regression not resolution.

## **Claim 2**

“Dr. Miranda’s findings were not clearly articulated in the original publication and therefore necessitated him putting pen to paper to draw attention to this fact.”

## **Fact**

Dr. Miranda’s reasons for “putting pen to paper” were not as Drs. Magnusson and Deva purport to represent, but for an entirely different reason. In Dr. Miranda’s own words about our paper,

“I read your article. It is very interesting and I agree with the description of the pathologic findings; please find attached a figure from the fluid in April 2015. I think the discrepancy is in the conclusions we can extract from the data. Your discussion brings interesting perspectives on the incidence of the disease, and how we can apply to our cases. I am going to pencil a letter to the editor with Mark Clemens, and emphasize that I agree with the pathologic discussion, but will mention that the conclusions do not follow to say by my experience. Overall, I hope our letter will enhance our discussion.” [7]

and

“As I mentioned you before, I completely agree with your interpretation. Your (resolution) case raises several questions, and the only way of answering is through the evaluation and follow up of more cases.” [8]

## **Claim 3**

Drs. Magnusson and Deva state, “These women (with Stage 1a disease) have a positive seroma tap for BIA-ALCL, but most have negative histology at the time of surgery, and there is no residual fluid left for cytology. With delays of up to 6 weeks prior to definitive capsulectomy after diagnosis, the failure to find tumor at resection does not prove regression or resolution. Both these patients presented as regression/resolution fall into this category, and so this report does not add anything novel to our understanding of BIA-ALCL as it currently stands.”

## **Fact**

As is carefully explained in the paper, the case of regression avoided an aspiration for 8 months after the onset of the seroma. Once a delayed confirmed diagnosis could be made, she declined surgical treatment for a further 3 months. During this time, repeated analysis of recurring seroma fluid demonstrated a progressive decrease in tumor burden and seroma volume. We note these findings are entirely consistent with the definition of regression upon which Drs. Magnusson and Deva rely.

In the resolution case, we refer Drs. Magnusson and Deva to our response to Dr. Miranda and Clemens [4] and merely reiterate here the fact that there was indeed “residual fluid left for cytology” present at explantation, and no tumor was present either in that fluid or in the capsule.

## **Claim 4**

Dr. Magnusson or Deva states “*It is not clear if Dr. Miranda was asked to review the second specimen, but I* (whether this is Dr. Magnusson or Deva writing here is unknown) *would encourage the authors to do so, to ensure accuracy of the diagnosis and to ensure that tumor was not missed*.”

## **Fact**

To the contrary, the paper does make this clear, “The authors wish to thank Dr. Roberto Miranda at the MD Anderson Cancer Centre, for his review of the slides of both cases.” All of the cytology and histology pathology materials were provided to Dr. Miranda.

## **Claim 5**

Drs. Magnusson and Deva question the motivation of the authors, “The question is then why are these strong claims being made on the basis of two case reports?”

## **Fact**

No claims of any nature were made solely “on the basis of two case reports.” As was clearly stated in the paper, “New observations and interpretation of the epidemiology of BIA-ALCL are made. These are supported by the presentation of two cases.”

## **Claim 6**

“Is there an underlying motivation for trying to downgrade a WHO listed cancer into a benign condition?”

## **Fact**

Ethically, we are motivated to ensure the WHO classification [9] continues to be updated to accurately reflect the totality of the evidence now available regarding the disease.

If that evidence shows a lymphoproliferative as well as a malignant version of the condition exists, then the current classification is wrong. As a consequence, lymphoproliferative patients may be being told incorrectly; they have a malignancy when in fact they do not. As was stated in the paper, this does not change the current requirement for surgery to confirm with certainty that there is no invasion. Notwithstanding, as we suggested in our response to Drs. Miranda and Clemens [4], this **does** change what we should tell patients with positive cytology and negative radiology.

We note that Dr. Deva has expressed contradictory opinions about our paper to different audiences. He writes with Magnusson “this report does not add anything novel to our understanding of BIA-ALCL as it currently stands.” Yet when commenting on the same paper independently of Dr. Magnusson, he says the opposite. In his role as a BIA-ALCL session moderator at an upcoming international conference, he has written, “*Great to see Daniel’s* (Fleming) *paper included in the session. I have had the pleasure of reviewing it and feel that it does raise some interesting questions that need to be answered in and around the epidemiology of BIA*-*ALCL*” [10].

Regarding Drs. Magnusson and Deva’s comments about conflict of interest, we reiterate that all of our conflicts of interest have been declared.
